# Selection of mutant µplasmin for amyloid-β cleavage in vivo

**DOI:** 10.1038/s41598-020-69079-8

**Published:** 2020-07-21

**Authors:** Dongying Yang, Wei Zhu, Yingjie Wang, Fangmei Tan, Zhiping Ma, Jiali Gao, Xinli Lin

**Affiliations:** 1 Shandong Provincial Key Laboratory of Biophysics, Shandong Key Laboratory in University of Functional Bioresource Utilization, School of Medicine and Nursing, Dezhou University, Daxuexi Road 566#, Dezhou, 253023 Shandong China; 2 Key Laboratory of State Ethnic Affairs Commission for Biological Technology, College of Life Science, South-Central University for Nationalities, Wuhan, China; 3 Institute of Systems and Physical Biology, Shenzhen Bay Laboratory, Shenzhen, 518132 China; 4 Department of Chemistry and Supercomputing Institute, University of Minnesota, Minneapolis, MN 55455 USA

**Keywords:** Biochemistry, Biotechnology, Computational biology and bioinformatics, Drug discovery, Neuroscience, Structural biology, Diseases

## Abstract

One of the main culprits of Alzheimer’s disease (AD) is the formation of toxic amyloid-β (Aβ) peptide polymers and the aggregation of Aβ to form plaques in the brain. We have developed techniques to purify the catalytic domain of plasmin, micro-plasmin (µPlm), which can be used for an Aβ-clearance based AD therapy. However, in serum, µPlm is irreversibly inhibited by its principal inhibitor α2-antiplasmin (α2-AP). In this study, we engineered and selected mutant forms of µPlm that are both catalytically active and insensitive to α2-AP inhibition. We identified surface residues of μPlm that might interact and bind α2-AP, and used an alanine-scanning mutagenesis method to select residues having higher activity but lower α2-AP inhibition. Then we employed saturation mutagenesis for further optimize both properties. Modeled complex structure of µPlm/α2-AP shows that F587 is a critical contact residue, which can be used as a starting position for further investigation.

## Introduction

Pathologically, Alzheimer’s disease (AD) is defined and mainly characterized by extracellular plaques and intracellular neurofibrillary tangles^1^. The extracellular plaques are primarily composed of amyloid-β (Aβ) peptides, and the intracellular neurofibrillary tangles are composed of the cytoskeletal protein tau^2,3^. Aβ is a mixed peptides of 40 and 42 residues, which is generated from amyloid-β precursor protein (AβPP) by the actions of two proteases, β-secretase (BACE-1) and γ-secretase^4^. Aβ oligomers are known to be cytotoxic, including directly causing neuron death through activation of ionotropic glutamate receptors^5^, causing microglial toxicity at low nanomolar concentrations^6^, disrupting neurotransmission^7^, and causing neuron-inflammation^8^. Results of nearly 30 years of research have mostly supported the amyloid cascade hypothesis^9,10^, stating that the overproduction of Aβ peptides (mostly from genetic defect), or the failure to effectively clear this peptide (most of the sporadic AD cases), leads to AD through Aβ toxicity and amyloid deposition. The latter is also thought to be involved in the formation of neurofibrillary tangles^11^. As a result, therapeutic research toward treatment of AD has mainly aimed at blocking production, hindering aggregation, or enhancing clearance of Aβ peptides^12^. One of the earliest Aβ-based therapeutic applications was immunotherapy using Aβ peptide as a vaccine^13^, although clinical toxicity has prevented further development of this strategy^14^. On the other hand, antibodies against Aβ peptides have been actively pursued as therapeutic agents; but with many failures of anti-Aβ antibody clinical trials, there have been times of pessimism in the field about the amyloid hypothesis^15^. Recently, however, the field has gained a renewed optimism due to a recent announcement of planed filing for market approval in the U.S. for aducanumab for BLA with the U.S. Food and Drug Administration (FDA) from Biogen Inc.^16^.

**Figure 1:**
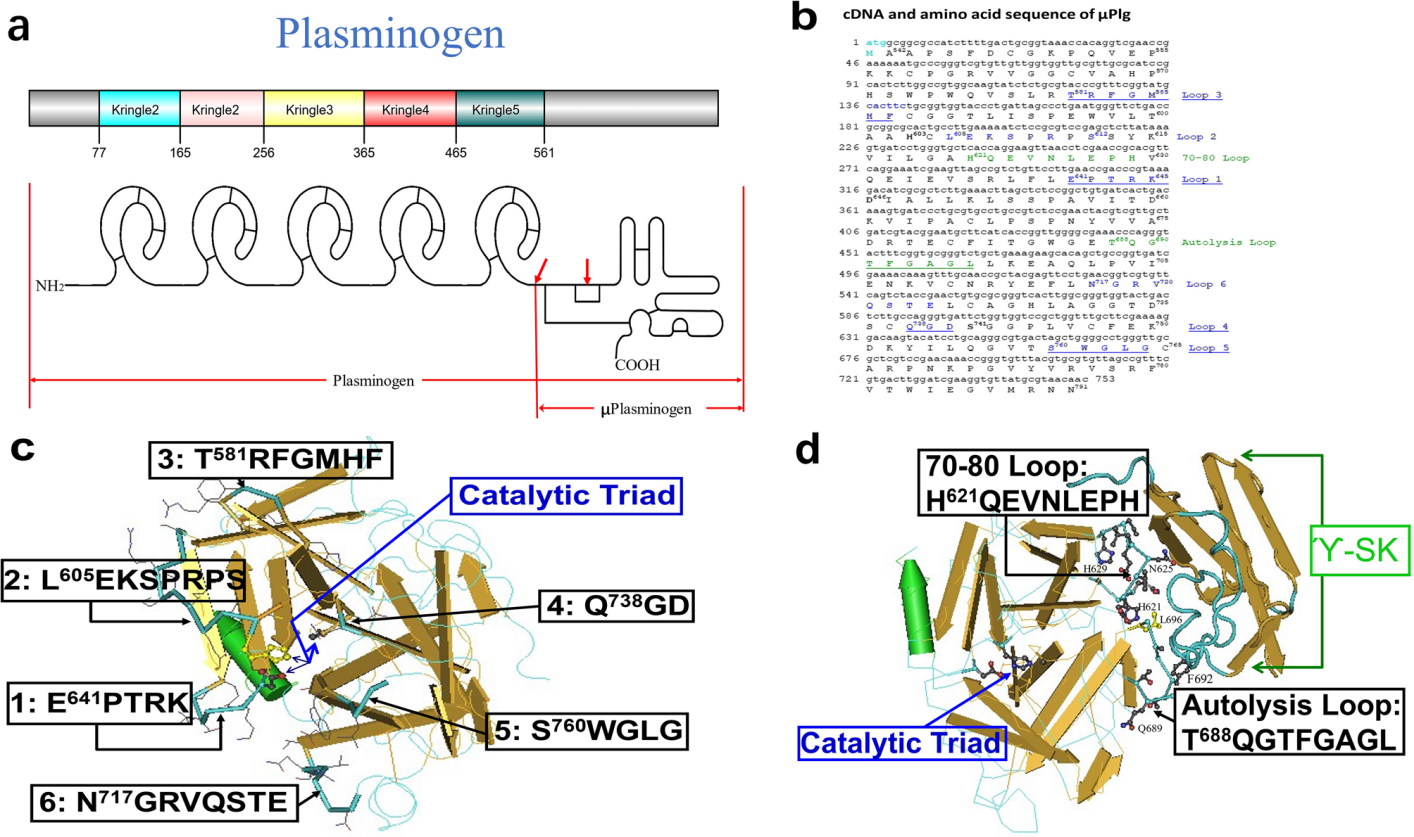
Structure-based design of alanine scanning mutagenesis. (**a**) Schematic presentation of Plg structure, indicating the 5 kringle domains and the catalytic domain (μPlg)^23^. (**b**) Synthetic gene and protein sequence of μPlg, with sequences of the loops labeled. (**c**) Loops 1–6 structure and residues around the active site catalytic triad of µPlm. The published µPlm structure^53^ were used. Numbering system is derived from the full length Plg^23^, and the amino acid number of the first amino acid in each loop is labeled. A total of 36 amino acids are labeled as the loop structure here. **d** Contact region between µPlm and the γ-domain of SK (labeled as ϓ-SK). A total of 18 amino acids are labeled as the loop structure here. Loop 7 is the 70–80 loop and loop 8 is the autolysis loop. In the structural presentation: the backbone, Wire; loops, Tubes with protein sidechains Wire; active side triad, Ball and Stick.

A large amount of data from many laboratories and clinics support the concept that an imbalance between the production and clearance of Aβ is a very early event in AD^9,17^. In normal physiological conditions, the Aβ production and clearance is a balanced biological process, including multiple actions of active and passive transport out of the brain, along with cell-mediated clearance, deposition into insoluble aggregates, and proteolytic degradation. Each of this process can act collectively or in concert to contribute Aβ catabolism. However, the overall research results have shown that proteolytic degradation is particularly important in regulating cerebral Aβ levels and AD pathogenesis. Saido and colleagues were some of the leading researchers to examine Aβ degradation in the living animal^18^. Subsequently more research showed that many proteolytic enzymes are involved in Aβ catabolism, including zinc-metalloproteases, cysteine proteases, and serine proteases (for review, see^17,19,20^), all of which have potential therapeutic values for treating AD. However, of all the proteases that are directly involved in degrading Aβ in vivo, only a recombinant form of plasmin (μPlm, see Fig. 1a) has been developed as a clinical drug^21,22^. Therefore, μPlm-based therapeutics can be a practical candidate for developing an enzyme augmentation therapy for Aβ degradation.

Plasmin (Plm) is the protease that digests fibrin, the main component of blood clots, in vivo^23^. Plm is the activation product of the inactive zymogen, plasminogen (Plg)^23^. All three functional proteases involved in plasmin-based thrombolysis are implicated in Aβ degradation: Plm, tissue-type plasminogen activator (tPA), and urokinase-type plasminogen activator (uPA). Of these, only Plm has been shown to directly degrade Aβ in both monomeric and fibrillar forms^24,25^, whereas tPA and uPA are believed to possess Aβ-cleavage function through Plg activation and the resulting action of Plm, the same as in thrombolysis in vivo^26^. In addition, it has been shown that plasmin can degrade and reduce the toxicity of both monomer and fibril Aβ^24,27^.

Studies in cultured cells have shown that purified Plm significantly decreases the level of neuronal injuries induced by aggregated Aβ^25,28^. In separate research, Ledesma et al. have not only shown that Plm degrades Aβ, but also shown consistently that the level of Plm is reduced in brain tissues from AD patients^29^. Other studies have also shown in vitro and in vivo correlation of Plm activity with Aβ level, and the possible therapeutic effect from increased Plm activity in vivo^30^. Despite of these activities, to our knowledge, the use of plasmin polypeptides as therapeutic agents to treat AD has not been reported.

One of the possible reasons is the extremely short serum half-life of Plm or its catalytic domain microplasmin (μPlm) (Fig. 1a). In serum, Plm quickly binds to and is inactivated by its principal inactivator, α2-Antiplasmin (α2-AP), and has a plasma half-life of only 0.2 s^31^. In order to overcome this technical barrier, we have used a structure-based mutagenesis approach to engineer α2-AP escaping mutants of μPlm toward a μPlm-based AD therapeutics. Although a longer half-life μPlm may cause side-effects such as bleeding with long-term chronic application required by AD therapy, our ultimate goal is to make a modified μPlm therapeutics that will be specific toward degrading Aβ peptide, but has no or much lower activity toward fibrin and other substrates, through structure-based “directional engineering” of the enzyme. Switching substrate specificity for serine proteases through protein engineering has been applied successfully^32^.

## Results

### Modeling of the molecular contact regions between µPlm and α2-AP

**Figure 2:**
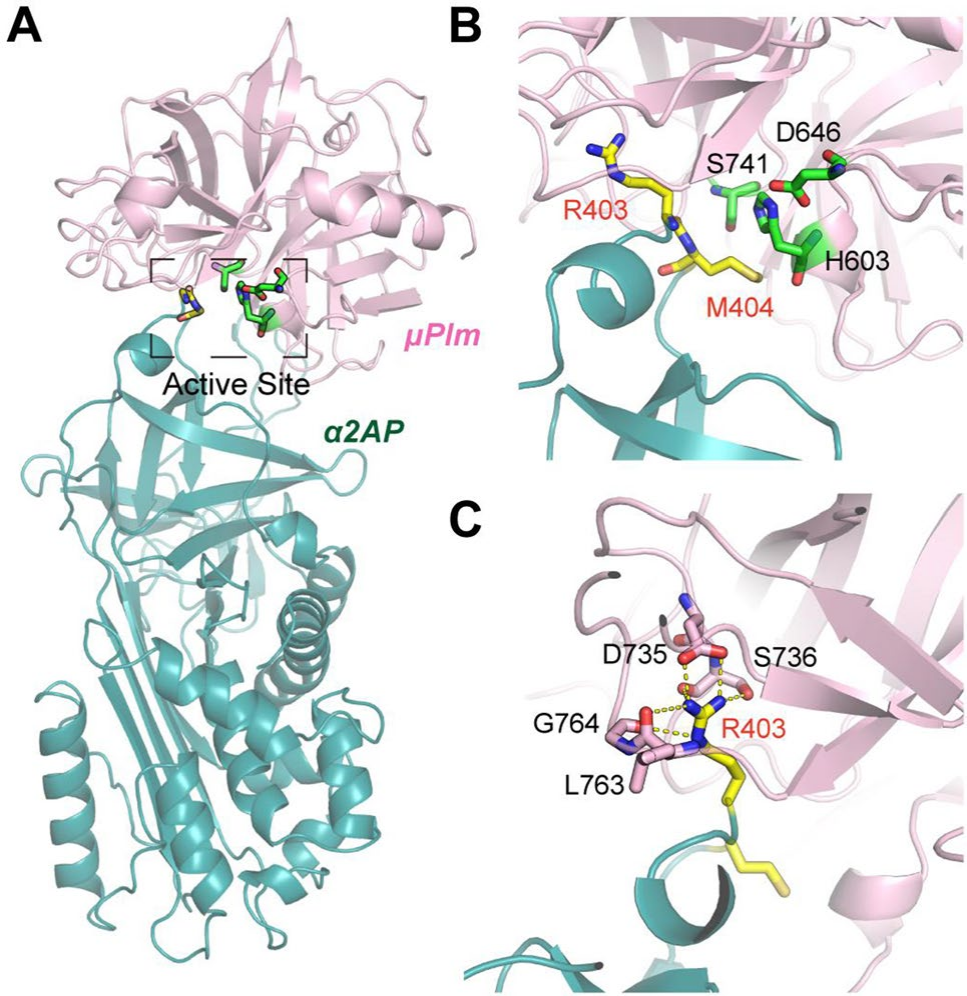
Stable complexes between µPlm and a2AP by homology modeling and subsequent MD simulations. (**A**) The overall architecture of the complex. (**B**) Detailed view of the active site, where the residues S741, H603 and D646 form the catalytic triad to initiate the covalent reaction with the backbone amide between R403 and M404. (**C**) Stabilization of the active site by extensive hydrogen bonds with the side chain of key residue R403.

A schematic presentation of the structure of Plg, μPlg, and structure-based mutagenesis design is shown in Fig. 1. In order to gain detailed structural insights on the plasmin:α2-AP complex, we superimposed the crystal structures for individual proteins to the Trypsin:antitrypsin crystal structure (PDB ID:1OPH) as a template^33^. Figure 2 illustrates that the interfacial interactions between α2-AP and µPlg is located at the loop of α2-AP that is docked into the active site of µPlm, where the backbone amide between R403 and M404 is the reaction site in the neighboring of the catalytic triads, H603, D646 and S741, with the distance of S741 to the backbone amide of ~ 4 Å, indicating a pre-attacking pose (Fig. 2B). The active site is maintained by an extensive network of hydrogen bonds that include the side chain of R403, D735 and S735 along with the backbone carbonyl groups of L763, G764, and S736 (Fig. 2C).

**Figure 3:**
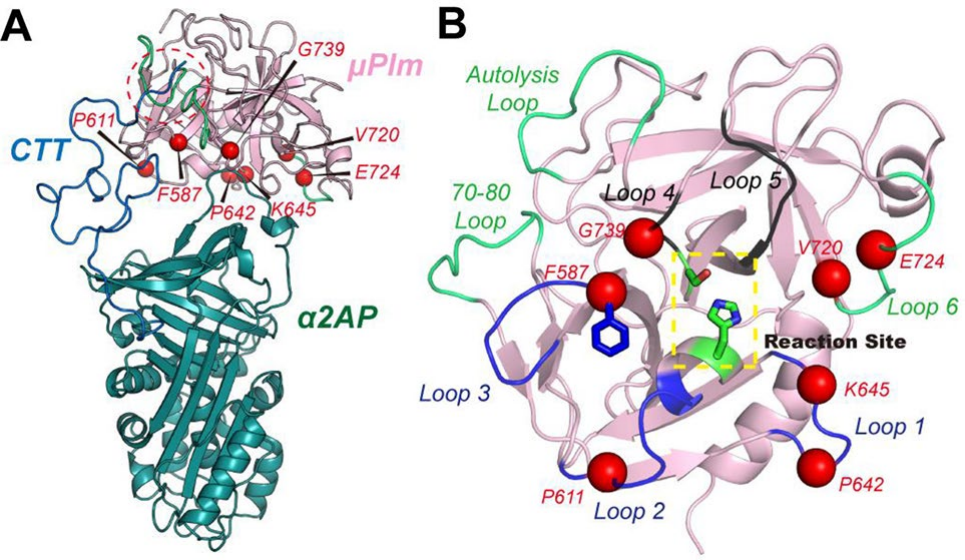
Distinct mutational effects mapping onto the interface. (**A**) Illustration of the interfacial loops and their mutational effects, where mutations on Loops 1–3 (highlighted in lightpink) exert moderate perturbation to the active site, with the exception of F587 (highlighted in hotpink), mutations on Loops 4–5 (highlighted in black) renders the protein inactive, and mutations on other distant loops (highlighted in hotpink) yield the desired perturbation. (**B**) Possible secondary interaction between the disordered C-terminal tail of a2AP with the autolysis loop and 70–80 loops of mPlm, interpreting the mutational effects of these two loops.

We further mapped the alanine scanning mutagenesis results (shown below) onto the complex structure to help interpret the effects of these peripheral loops on the active site. Figure 3A indicates that the effects of loop mutations can be classified into three categories: (I) loops 4 and 5 that are closest to the active site, whose mutations completely abolished the enzyme activity (II) loops 1–3 that are further apart whose mutations has moderate effects on catalytic activity and the IC50, except F587, which in fact has the largest desired perturbation, and (III) loops 6, the 70–80 loop and autolysis loop, which are distant from the active site, but their mutations dramatically increase IC50, suggesting that a secondary interaction between the plasmin and α2-AP might be important to stabilize the complex. The missing C-terminal tail (CTT) in the crystal structure might provide such fuzzy and transient interactions. Indeed, molecular dynamics (MD) simulation lasting 100 ns show that the CTT is flexible and can have transient interactions with the neighborhood µPlm, and these secondary, non-specific interactions may explain the surprising effects of alanine mutations on these distant loops.

### Expression and purification

**Figure 4:**
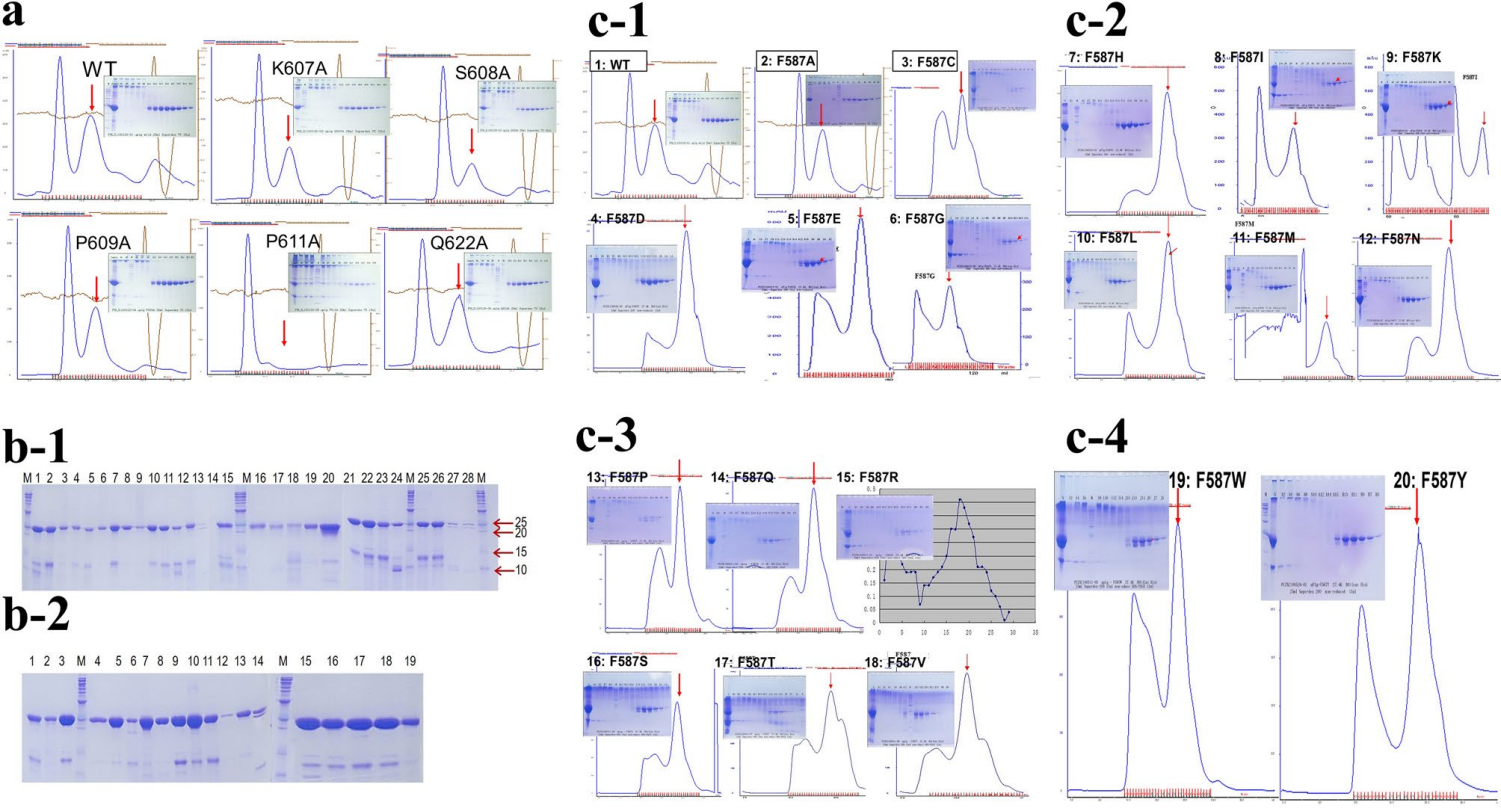
SDS-PAGE and Superdex 75 profiles. After refolding and concentration by ultrafiltration, µPlg wild-type and mutants were purified on a Superdex 75 column as described^47^. (**A**) An example of the wild-type and 5 alanine mutants. In the SEC graph, the first peak is the unfolded aggregates, and the second peak (red arrows) is the refolded peak. A non-reduced SDS-PAGE of the purified proteins is shown in the insert. (**b-1**) SDS-PAGE of 26 samples of the purified alanine mutants along with wild-type human and mouse samples. M is a molecular weight marker. 1. wild type; 2. W761A; 3. G762A; 4. R719A; 5. G695A; 6. G739A; 7. G739A; 8. G739A; 9. G764A; 10. T688A; 11. T688A; 12. L626A; 13. G693A; 14. K645A; 15. V624A; 16. H621A; 17. T691A; 18. V720A; 19. R610A; 20. Mouse-wild; 21. G690A; 22. L696A; 23. D740A; 24. F692A; 25. S760A; 26. L763A; 27. R582A; 28. P642A. (**b-2**) SDS-PAGE of purified F587 mutants. 1. F587H; 2. F587I; 3. F587K; 4. F587L; 5. F587M; 6. F587N; 7. F587P; 8. F587Q; 9. F587R; 10. F587S; 11. F587T; 12. F587Y; 13. F587W; 14. F587V; 15. Wild-type (F); 16. F587C; 17. F587D; 18. F587E; 19. F587G. (**c**) (**c-1**–**c-4**) are Superdex 75 purification profile and SDS-PAGE of F587 saturation mutagenesis proteins. (**c-1**) 1. Wild-type; 2. F587A; 3. F587C; 4. F587D; 5. F587E; 6. F587G. (**c-2**) 7. F587H; 8. F587I; 9. F587K; 10. F587L; 11. F587M; 12.F587N. (**c-3**) 13. F587P; 14. F587Q; 15. F587R; 16. F587S; 17. F587T; 18. F587V. (**c-4**) 19. F587W; 20. F587Y. Full-length gels are presented in Supplementary Fig. 4.

**Figure 5:**
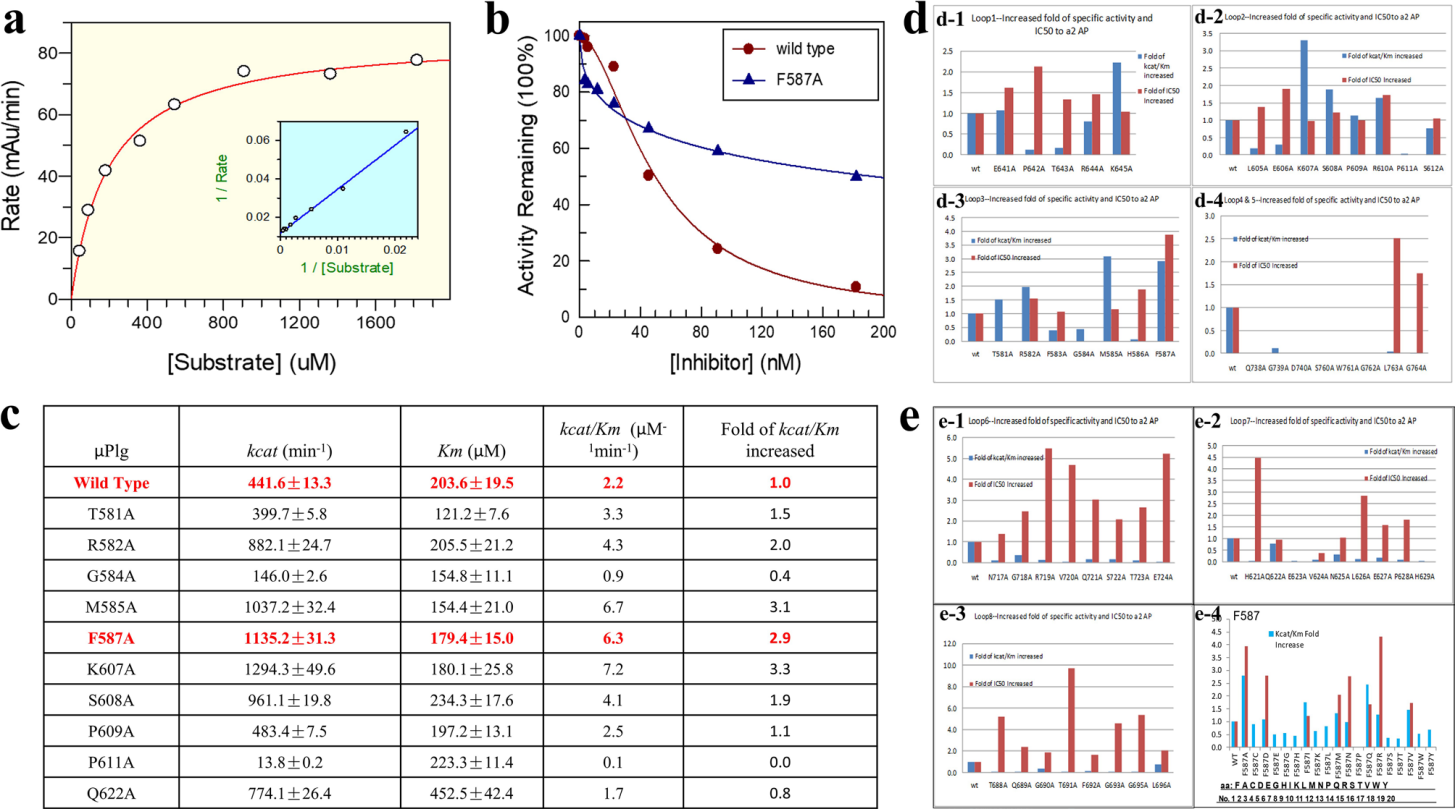
Kinetics and α2-AP inhibition. (**a**) An example of Michealis–Menten kinetic measurement of wild-type µPlm. (**b**) Comparison of α2-AP inhibition of wild-type and F587A, shows the reduced inhibition of F587A by α2-AP. (**c**) Kinetic parameters of loop 2 and 3 alanine mutants, showed the increased catalytic efficiency (*Kcat/Km*) of F587A. (**d**) Kinetic parameters and α2-AP inhibition of loops 1–5. Blue bars represent fold of catalytic efficiency, and the red bars represent fold of IC50 relative to the wild-type. (**d-1**) loop 1; (**d-2**) loop 2; (**d-3**) loop 3; (**d-4**) loops 4 and 5. (**e**) Same as (**d**), with loops 6–8 and F587 saturation mutants. (**e-1**) Loop 6; (**e-2**) loop 7; (**e-3**) loop 8; (**e-4**) F587 saturation mutants, including all 20 amino acid changes.

As the first step, we selected 54 residues shown in Fig. 1c,d for alanine scanning mutagenesis. After cloning and expression, a total of 52 mutants were successfully refolded and purified, as shown in Fig. 4, which shows some of the size exclusion chromatography (SEC) purification profiles and non-reduced SDS-PAGE of purified mutants. We then tested for kinetics and α2-AP inhibition for the purified proteins. Of these, 45 have significant activities (Fig. 5), and the purification and SDS-PAGE of 27 of the purified mutants are shown in Fig. 4a,b-1. From the results of the kinetics and inhibition studies (Fig. 5), we selected F587 for saturation mutagenesis. All of the designed F587 mutants were successfully expressed and purified, with SDS-PAGE for 18 of the mutants shown in Fig. 4b-2, and the Superdex 75 purification profiles of all of the 20 mutants shown in Fig. 4c. Thus, we purified a total of 71 mutants, and obtained kinetics and inhibition data for 67 of the samples. Figure 4 shows that the wild-type (WT) and mutant μPlg can be purified with a one-step SEC purification after refolding. This simplified purification procedure allows for the high-throughput screening of µPlm mutants.

### Kinetics and α2-AP inhibition

We first performed standard Michaelis–Menten kinetic measurements for the wild-type and all purified mutants, with examples of wild-type and F587A mutant shown in Fig. 5a,b. We then performed α2-AP inhibition studies, using the wild-type enzyme as a reference for every set of experiment. An example of the inhibition curve for wild-type and F587A performed side by side is shown in Fig. 5b, which indicates clearly that at high concentration of α2-AP, the wild-type µPlm lost almost all of its activity, while the F587A mutant was still active, keeping almost half of its activity at the highest concentration of the inhibitor tested. An example of the inhibition data set on loops 2 and 3 is shown as a table in Fig. 5c, which exemplified the parameters measured with standard errors. The whole sets of measurement are shown in Fig. 5d,e in a bar graph: the error bars were not illustrated for clarity.

Figure 5d-1 shows the inhibition kinetics of Loop1 (Fig. 1c), corresponding to part of the “94-shunt” loop in µPlm (all the terminologies for structural definition are according to a reference^34^), which is connected to the active site D646. The relative catalytic efficiency (*kcat/Km*, fold increase to the wild-type) of P642A is decreased dramatically, but the IC50 value is enhanced by a factor of 2. The low activity of P642A is due to a large increase in Km. Although the relative catalytic efficiency of the K645A mutation is increased by twofold, IC50 remains unchanged. Figure 5d-2 shows the inhibition kinetics of Loop2, which corresponds to part of the “60-loop” in µPlm, extending out of the active site H603. The figure shows that the P610A mutation leads to higher catalytic activity and along with an increase in IC50. Figure 5d-3 exhibits the inhibition kinetics of Loop3, corresponding to the “37-loop” in µPlm. Four of the alanine mutants in loop3 have higher catalytic activities than that of the wild-type enzyme. The main goal of this study is to discover mutations that have enhanced catalytic activities, and at the same time, are resistant to or capable of “escaping” α2-AP inhibition. In all of the alanine mutants we have scanned, the F587A change is the best candidate. We therefore select the F587 position for saturation mutagenesis. Figure 5d-4 displays the inhibition kinetics of mutations in loops 4, which is the “oxyanion stabilizing loop” of µPlm, and loop 5, which consists of the “S1 entry frame” loop. Clearly, mutations both in loop 4 and loop 5 resulted in dramatic loss of catalytic activity. G739A shows a lower catalytic efficiency (fold of *kcat* = 0.6, *Km* = 4.9), but is not inhibited by α2-AP up to 400 nM. This finding indicates that additional research may be targeted at this position. The loss of catalytic efficiency of G739A is mainly caused by the increase of *Km.* Figure 5e-1 depicts the inhibition kinetics of amino acid alteration in Loop 6, which terms as the “methionine loop” in µPlm. We find that mutations in loop 6 also result in dramatic loss of catalytic activity, which may be attributed to increased Km, especially for V720A (fold of *kcat* = 0.1, *Km* = 3.3) and E724A (fold of *kcat* = 0.1, *Km* = 5.2). Figure 5e-2 illustrates the results by mutations of Loop 7, the 70–80 loop of the µPlm structure, which is also called the “Ca^2+^ binding” loop. Loss of catalytic acitivity is observed on alanine mutations in this loop, despite the fact that loop 7 is far away from the active site (Fig. 1). Figure 5e-3 features the activity profile of Loop 8, or the “autolysis loop” of µPlm, which are not active upon amino acid mutations, and thus are not prone to α2-AP inhibition.

Finally, we present the kinetics of saturation mutations at the F587 position in Fig. 5e4, in which five of the mutants gain catalytic activities and show greater resistance to α2-AP inhibition compare to the wild type. Of these mutants, F587A and F587R are identical to have the desirable properties which may be further studied for in vivo activities.

### Covalent attachment of human and mouse μPlm to α2-AP

Similar to the inhibition of other serine proteases by serpins, the inhibition of µPlm by α2-AP is a two stage process^35,36^. The first step is a fast and reversible second-order reaction, leading to a noncovalent 1:1 Michaelis-like complex of α2-AP with µPlm, and the second step is a slower and irreversible first-order reaction to form a covalent linkage between the α2-AP insertion loop and the catalytic Ser residue of µPlm. The “suicide” reaction effectively and permanently inactivates the serine protease µPlm.

**Figure 6:**
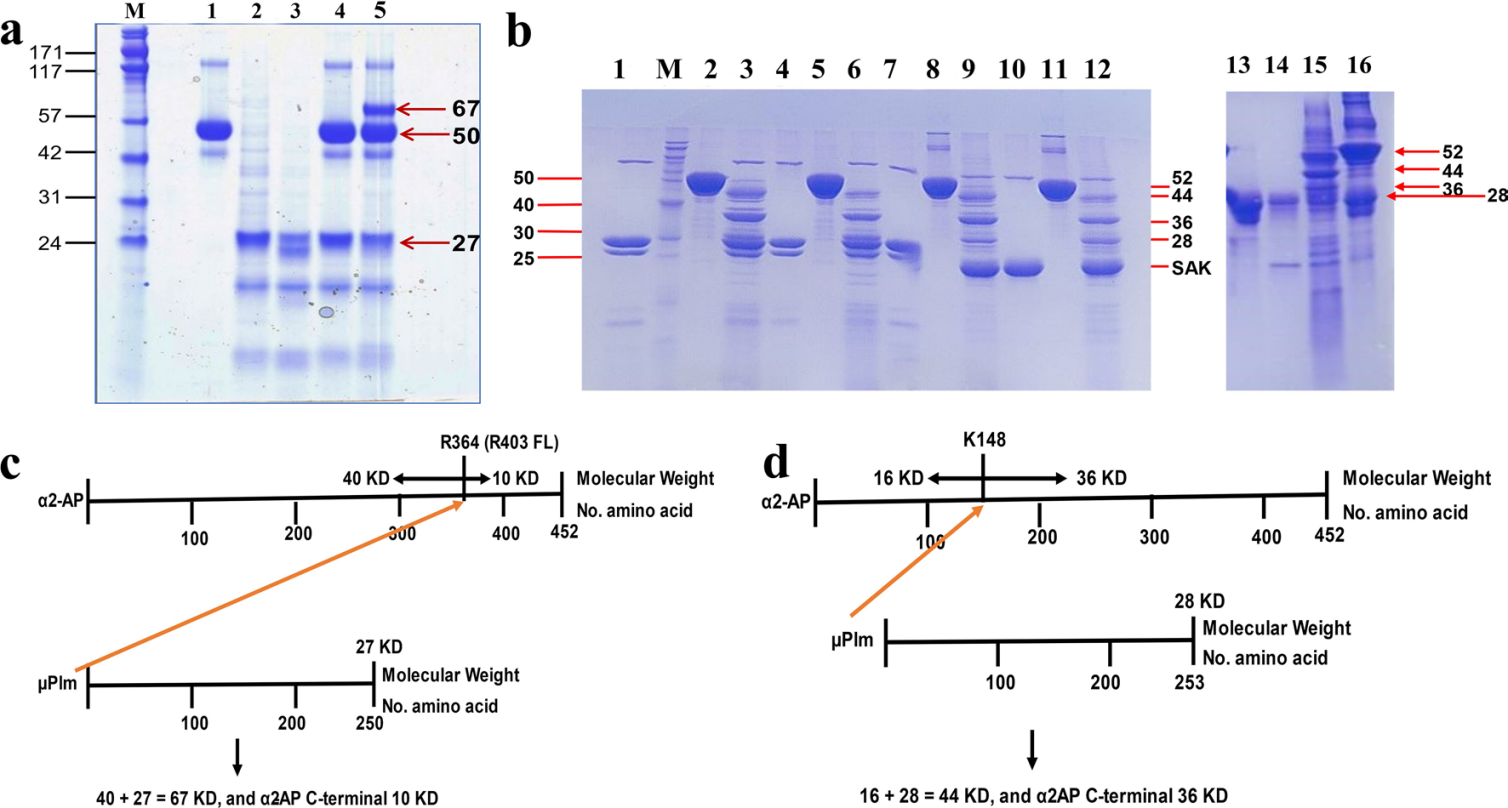
μPlm-α2-AP complex formation. (**a**) Human µPlm and α2-AP. M. Molecular weight marker, standard molecular weight is labeled on the left, and molecular weight (KD) of the protein fragments is labeled on the right. 1. Human α2-AP, 50 KD; 2. Human µPlg, 27 KD; 3, Human µPlm; 4, Human µPlg + α2-AP; 5, Human µPlm + α2-AP. (**b**) Mouse µPlm and α2-AP. Standard molecular weight is labeled on the left, and molecular weight (KD) of the protein fragments is labeled on the right. 1–6, reduced SDS-PAGE; 7–12, non-reduced SDS-PAGE. 1. Mouse µPlm, 28 KD; M, Molecular weight marker, labeled on the left; 2, Mouse α2-AP, 52 KD; 3, Mouse µPlm + Mouse α2-AP; 4. Mouse µPlm; 5, Mouse α2-AP; 6, Mouse µPlm + Mouse α2-AP; 7. Mouse µPlm; 8, Mouse α2-AP; 9, Mouse µPlm + Mouse α2-AP; 10. Mouse µPlm; 11, Mouse α2-AP; 12, Mouse µPlm + Mouse α2-AP. Reaction time, 1–3 and 7–9, 2 min; 4–6 and 10–12, 5 min. Urokinase (1:20) was used to activate µPlg in lanes 3, 4, 6, and 7; while staphylokinase (SAK, 18.5 KD, 1:1) was use to activate µPlg in lanes 9, 10, and 12. Lanes 13–16 were performed in a separate experiment. 13, Mouse µPlg; 14, Mouse µPlm; 15, Mouse µPlm + Mouse α2-AP; 16, Mouse µPlg + Mouse α2-AP. (**c**) Schematic presentation of the reaction between human α2-AP and µPlm shown in Fig. 4a. The red arrow from µPlm to R364 of human mature α2-AP illustrate the nucleophilic attach of the active site serine of µPlm toward the α2-AP substrate at the R364 (P1) position; (**d**) schematic presentation of the reaction between mouse α2-AP and µPlm shown in Fig. 4b. The red arrow from µPlm to K148 of α2-AP illustrate the nucleophilic attach of the active site serine of µPlm toward the α2-AP substrate at the K148 (P1) position. Full-length gels are presented in Supplementary Fig. 6.

The SDS-PAGE of recombinant human µPlg, µPlm, α2-AP separately and their reaction mixtures are shown in Fig. 6a. In a reaction mixture containing human µPlg and α2-AP, there is no reaction observed (lane 4), confirming that the zymogen has no catalytic activity. On the other hand, the reaction mixture of µPlm and α2-AP gives a covalent complex of 67 KD (40 KD N-α2-AP + 27 KD µPlm), shown in Fig. 6a and schematically in Fig. 6c, confirming covalent bond formation between R364 of α2-AP and the catalytic serine in the active site of µPlm^35^.

The reaction between mouse µPlg and α2-AP is presented in Fig. 6b, and schematically illustrated in Fig. 6d, which illustrates the 52KD mouse α2-AP protein (464 amino acids) and the 28 KD mouse µPlm (253 amino acids). After the reaction, the inhibitory process left a stalled band of 44 KD adduct and a 36 KD fragment in Fig. 6b, as a result of covalent attachment at the K148 site of α2-AP to µPlm. In some of the reactions, the mouse µPlg can be activated by SAK, shown in lanes 9, 10, 12 in Fig. 6b, but lane 16 clearly shows that mouse µPlg does not cleave α2-AP, consistent with the notion that the catalytic activity originates from the mouse µPlm.

## Discussion

**Figure 7:**
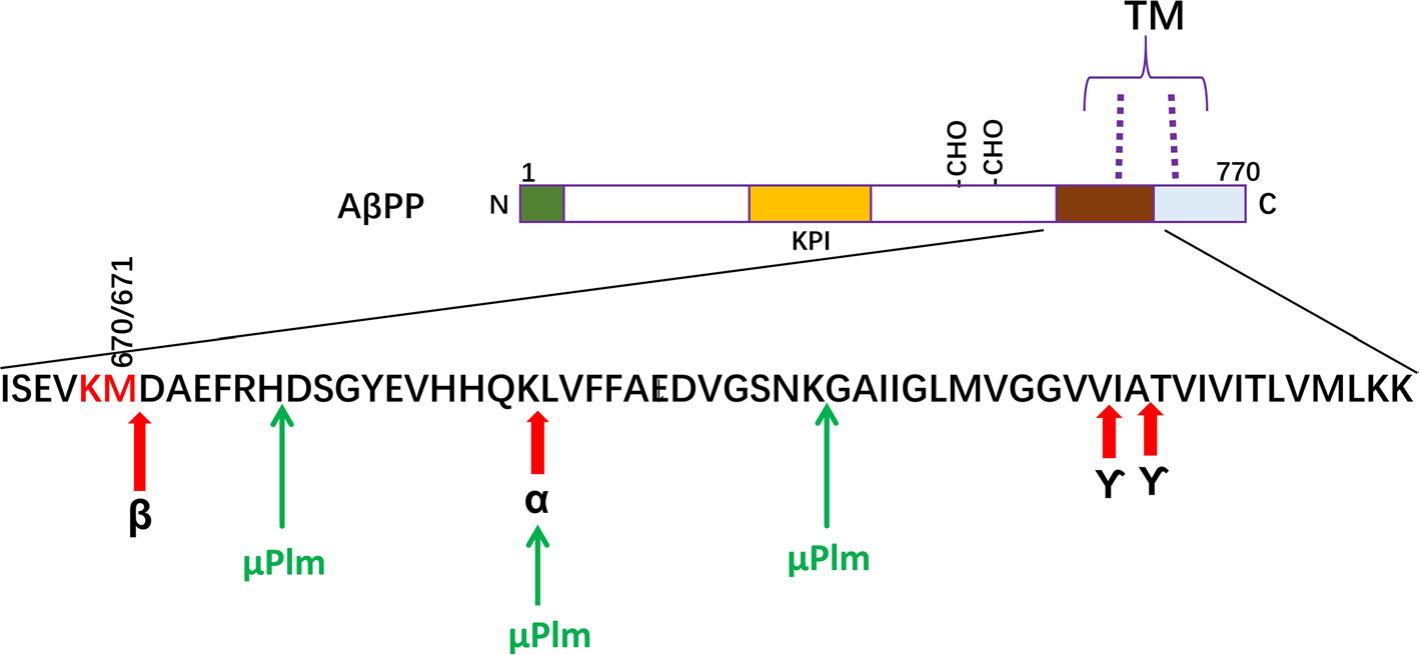
Schematic presentation of the protein structure of AβPP and the Aβ peptide processing sites. The α-secretase, β-secretase, and γ-secretase cleavage sites are indicated. The figure shows that μPlm cut Aβ at 3 basic residues as the P1 position, the same as native plasmin^24,25^. *TM* transmembrane domain, *KPI* Kunitz Protease Inhibitor domain, *CHO* glycosylation sites.

Following reactions of the present recombinant µPlm with an Aβ-40 peptide, we found through LC–MS analysis that Aβ-40 was cleaved at the three expected plasmin sites (P1 positions are bold and underlined): DAEFR**H**DSGY EYHHQ**K**LVFF AEDVGSN**K**GA IIGLM VGGVV. Figure 7 summarizes a schematic presentation of the peptide structure, and the cleavage of AβPP by β and γ-secretases to generate Aβ-40 and Aβ-42 peptide, in which Aβ-42 is particularly neuron toxic. In practical situation, cleavage at just a single site is sufficient to detoxify Aβ-42 completely.

Most of the Aβ clearance clinical drugs pipelines are based on Aβ-specific monoclonal antibodies (Mabs)^12,15^. Although past research has identified many different kinds of proteolytic enzymes involved in Aβ catabolism^31^, to our knowledge, however, there has been no therapeutic development based on proteolytic clearance of the Aβ peptide^15^. On the other hand, there are significant advantages for using enzymatic Aβ cleavage in comparison with the current Mab clearance method. First, one antibody binds and removes only one set of Aβ peptide, while one proteolytic enzyme can hydrolyze many. Therefore, a therapeutic enzyme may be more efficient than a Mab. Second, even humanized Mab is artificial and ‘foreign”, while therapeutic μPlm is “native” and serves as a simple “replacement” of what is deficient in vivo. Thus a properly engineered therapeutic μPlm may generate less side effects in long–term application. Third, the *E.* *coli* produced therapeutic μPlm is much less expensive to manufacture than the mammalian cell produced Mab, an important aspect considering the raising health care costs and large population of AD patients.

As described above, in order to develop a μPlm-based Aβ cleavage therapeutics, it is necessary to engineer recombinant μPlm that can escape α2-AP inhibition. As a first step toward this goal, we have modeled the contact region between μPlm and α2-AP (Figs. 2, 3). On the basis of structural modeling, we identified surface residues around the active site of μPlm that might interact with α2-AP (Fig. 1c). In addition to the loop structures in Fig. 1c, Esmon and Mather^32^ found that the γ-domain of streptokinase (SK) (γ-SK) in the autolysis contact region forms a major topologic collision, preventing α2-AP from binding to the SK-µPlm complex. That study helps to explain the insensitivity of SK-µPlm to α2-AP inhibition^32^. Thus, besides the loop regions around the active site (Fig. 1c), the autolysis loop region and the calcium binding loop may also be involved in α2-AP recognition^32^. Docking of µPlg into the SK-µPlm active site indicates that γ-SK involved little structural interaction with substrate binding^32^, implying that mutations in this region may not only disrupt α2-AP binding, but also have minimum interference with substrate binding and catalytic activity. We therefore decided to include these two regions in selection for alanine-scanning mutagenesis studies (Figs. 1d, 3B). Since the γ-domain of SK can block α2-AP binding without interfering with µPlm activity, an important implication from the analysis is that the use of a non-specific substitution such as polyethylene glycol (PEG) for γ-SK may have similar structural effect.

PEGylated drugs are known to extend in vivo half-life, reduce or eliminate immunogenicity^37^, and have already been approved by the FDA^38^. We envision a two-step process for Cys-PEGylation screening at selected residues in loops 7 and 8 where γ-SK binding is located. First, we may identify residues according to preliminary alanine scanning mutagenesis results for Cys mutation and characterize the kinetic properties of the resulting Cys mutants. For example, we may select Q622 and L626 of Loop 6 (Fig. 5e-2) and G690 and L696 of Loop 7 (Fig. 5e-3) as our initial Cys mutation studies. Second, for Cys mutants that have desired kinetic properties, we may perform Cys-PEGylation and characterize the PEGylated μPlm (PEG-μPlm) mutants. Structurally (Figs. 1c, 3B), using polyethylene glycol to functionally “replacing” the γ-domain of SK is a rational approach, and the implication of the resulting PEGylated μPlm can be far reaching in all aspects of drugable properties: native activity, blocking α2-AP inhibition, reducing or eliminating possible immunogenicity, and longer in vivo half-life.

To identify the residues in the loops that could potentially escape α2-AP inhibition, we screened all of the 54 loop residues identified in Fig. 1c,d, using an alanine-scanning mutagenesis method^39^. We expressed and successfully purified most of the mutants using our method (Fig. 4), and performed kinetic studies (Fig. 5). The results enabled us to identified F587A for additional saturation mutagenesis studies, revealing that F587A and F587R are the best candidates, balancing catalytic efficiency and avoidance of α2-AP inhibition (Fig. 5c,e4). It would be of interest to examine cooperative effects of multiple mutations in future investigations.

For the selected mutants, further testing of the catalytic efficiency toward fibrinogen, fibrin, and Aβ substrates will be performed to select mutants that has high activity toward Aβ-42, insensitive to α2-AP inhibition, and low activity toward fibrinogen and fibrin.

The present study provides a further understanding of the kinetic and thermodynamic interactions between μPlm and α2-AP, an important biological process has not been well studied. In addition, because any potential AD drug, including the one investigated in this study, will likely be used for long-term chronic application, it is desirable to design an animal model for long-term efficacy and toxicity test. However, using human µPlm for long-term testing in a mouse model may not be suitable due to host differences and potential immune rejection. We therefore cloned a mouse version of µPlm and α2-AP, and studied the reaction mechanism and inhibition interactions (Fig. 6b). It is known that α2-AP reacts with µPlm to form an enzymatically inactive stoichiometric 1:1 complex, with an initially fast second order reversible reaction followed by a slower first order irreversible covalent reaction^35,40^. As shown in Fig. 6, both human and mouse µPlm react with their α2-AP to form a covalent complex. Detailed analysis of the mechanism will be performed and published separately. Other than scientific values, the successful construction of mouse µPlm-α2-AP system formed a basis for pre-clinical development of the µPlm based AD therapeutics.

**Figure 8:**
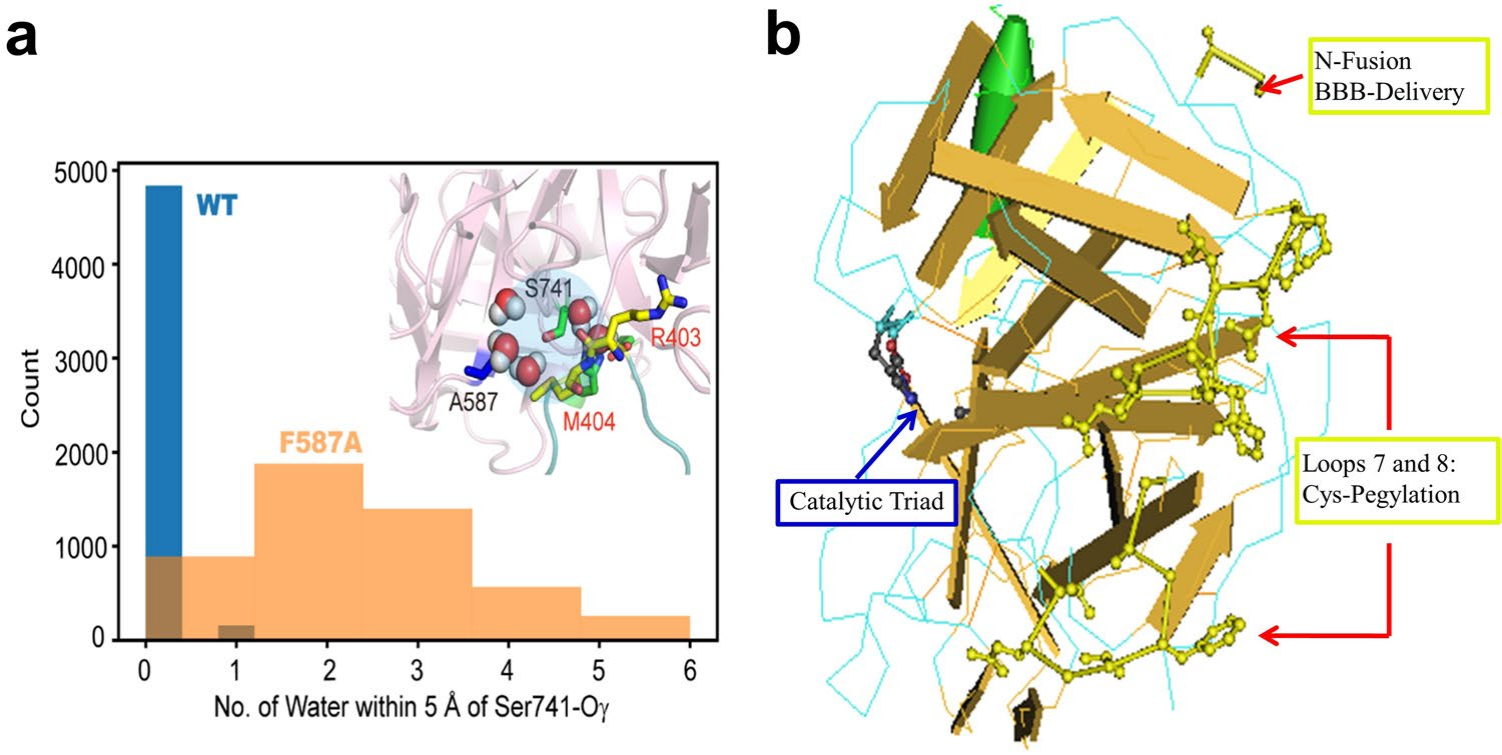
F587A structural features (**a**) and the N-terminal fusion and Cys-pegylation positions of μPlm (**b**). (**a**) Comparison of water distribution around the active site between WT and F587A mutant, with the inset figure highlighting the multiple water molecules around S741 for F587A mutant. (**b**) The figure shows that the N-terminal is on the opposite side of the catalytic triad and there is no steric hindrance for connecting a fusion partner to the N-terminal of μPlm (also see Fig. 1a).

To provide further insight into the origin that the F587A mutation remains active, but is also resistant to inhibition by α2-AP, we carried out molecular dynamic (MD) simulations of both the wild-type and mutant enzymes. Notably, we observed that the distribution of water molecules in the active site is affected following the replacement of a bulky hydrophobic residue F587 by alanine. Whereas water molecules are infrequently observed in the active site of the WT enzyme, MD simulation shows that on an average 2–3 are located in the active site of the mutant (Fig. 8a). For the WT enzyme, inhibition of µPlm by a2-AP is achieved by forming a covalent complex. On the other hand, in the F587A mutant, water molecules in the active site can act as a generalized base and initiate nucleophilic attack on the carbonyl group of the peptide bond to trigger bond cleavage and release of the bonded or “trapped” a2-AP, escaping the inhibition. Furthermore, for other substrates, favorable access of water molecules in the active site can facilitate the proteolysis reaction, and enhance catalytic efficiency. A more in-depth investigation of the reaction mechanism through combined quantum mechanical and molecular mechanical (QM/MM) simulations and experimental verifications will be reported in a forthcoming study.

Another key issue is the difficulties for macromolecules to cross the blood brain barrier (BBB), which has hindered the development of neurotherapeutic agents, especially large molecule drugs for CNS diseases^41^. To facilitate the BBB crossing, we may use fusion approaches connecting μPlm to peptides or single-chain Mab (scMab) that function as a receptor-mediated transcytosis carrier. On the other hand, it has been shown that Aβ peptides cross the BBB in AD patients. Research has shown that soluble, blood-born Aβ peptides can cross a defective BBB and interact with neurons in the brain^42^, which indicates that lowering the serum concentration of Aβ peptides by μPlm therapeutics may tipping the balance of Aβ in the CNS. Furthermore, it has shown that Aβ peptides may across the intact BBB through the low density lipoprotein receptor related protein 1 (LRP1) mediated cellular uptake^43^. These results suggest that the blood may serve as a major, chronic source of soluble, exogenous Aβ peptides that can cross BBB and bind selectively to certain subtypes of neurons and accumulate within these cells^17,42^. In addition, it has been shown that peripherally applied Aβ-containing inoculate induced cerebral β-amyloidosis^44^, further implying that clearing peripheral Aβ may be as important as cerebral clearance. Because of this, a μPlm-based fusion approach may clear both peripheral and cerebral Aβ, further strengthening the therapeutic efficiency. For this purpose, we may design a “molecular Trojan horse” strategy^45^ by engineering fusion partners to ferry the therapeutic μPlm across the human BBB. Different fusion partners with proven receptor mediated BBB transcytosis properties can be designed in order to select a highly efficient transporter for our selected μPlm construct, as shown in Fig. 8b.

The overarching significance of the study is that if the desired μPlm construct is developed, it may serve as a radically new AD therapeutics that is more efficient, less toxic, less or not at all immunogenic, inexpensive to manufacture, and with broader application potential. Beyond AD application, a mutant μPlm developed by “directional engineering” may provide a more effective alternative therapy for indications such as pulmonary embolism, deep vein thrombosis, peripheral arterial thrombosis, stroke, and atrial fibrillation^46^.

## Material and methods

### Genes and cloning

A synthetic gene of human μPlg optimized for *E.* *coli* expression was the same as previously published, and mutagenesis for Plg were also routinely performed in our lab as published^47^. Mouse μPlg (GENE ID: 18815) was cloned, expressed, refolded, and purified in a similar method as human. Mature human and mouse alpha-2 antiplasmin (HAP, NM_000934.3; MAP, GC-Mm04554), plasminogen activators pro-urokinase (GENE ID: AAA61253), streptokinase (SK) and staphylokinase (SAK)^48^ were also cloned into a pET-11 expression vector for *E.* *coli* expression.

### Microplasminogen (μPlg) mutagenesis design and method

A schematic presentation of the structure of Plg, μPlg, and structure-based mutagenesis design is shown in Fig. 1. The designed μPlg WT and mutants were constructed, expressed, refolded, and purified as described^47,49^.

### Expression, inclusion body refolding, and purification

The sequence verified mutant plasmids were transformed into *E.* *coli* strain BL21(DE3) for expression, refolding, and purification following the same procedure as previously described^47^. Briefly, *E.* *coli* containing the expression plasmids were expressed in a high-density shaker flask auto-induction system^50^. The broth was then spun down and the pellet was washed extensively and put through freeze thaw cycles with lysozyme to purify the inclusion bodies (IB). The purified inclusion bodies were dissolved in an 8 M urea buffer [8 M urea, 0.1 M Tris, 1 mM glycine, 1 mM EDTA, 10 mM -mercaptoethanol, 10 mM dithiothreitol (DTT), 1 mM reduced glutathione (GSH), 0.1 mM oxidized glutathione (GSSG), pH 10.5 with a final concentration of 2 mg/ml]. The solution was rapidly diluted into 20 volumes of 20 mM Tris, 0.2 M l-arginine, pH 10.5. The pH of the solution was slowly adjusted to pH 8 with 6 M HCl as described^51^. The refolded protein was then concentrated by ultrafiltration, and purified by various types of column chromatography as described^47^. For initial screening, we grew 200 ml culture for the WT μPlg and each of the mutants, yielding about 200 mg of highly purified IB for each construct.

Human and mouse alpha-2 antiplasmin (HAP and MAP) were refolded in a buffer containing 20 mM Tris, 0.1 mM GSH, 0.01 mM GSSG, pH 9.0, and purified essentially the same as that of the μPlg. The methods for optimized expression and purification of pro-urokinase will be published elsewhere. Streptokinase (SK) and staphylokinase were expressed and purified as published (High yielding recombinant Staphylokinase in bacterial expression system–cloning, expression, purification and activity studies^48^.

### Activation and kinetic measurements

Chromogenic substrate pGlu-Phe-Lys-pNA (S-2403) was from Chromogenix (Sweden). 4-Nitrophenyl 4-guanidinobenzoate hydrochloride (pNPGB) was from Aldrich. NUPAGE 4–12% BT GEL was from Invitrogen. Other chemicals and protein reagents were from SIGMA/Aldrich. Kinetic measurement was performed similarly as described^47^. Briefly, the refolded and purified μPlg zymogens (35.5 µM) were activated with a plasminogen activator such as urokinase (20:1) at 37 °C for 4 min in a reaction mixture containing 25 mM Tris–HCl, pH 7.4, 50 mM NaCl. The active site of the activated μPlm was titrated using pNPGB as described^52^. The activated zymogens were diluted to 5.5 µM, and then 10 µl was mixed with 100 µl of 0.0625 mM, 0.125 mM, 0.25 mM, 0.5 mM, 0.75 mM, 1.0 mM, 1.5 mM, or 2.0 mM of substrate S-2403 in the assay buffer (25 mM Tris–HCl, 50 mM NaCl, pH 7.4). The generation of amidolytic activity was monitored (at 405 nm) at 37 °C in 10 s intervals for 20 min using SpectraMax 250 microplate reader (Molecular Devices). The data was plotted as velocity vs. substrate using GraFit version 7 (Erithacus Software) and the V_max_ and K_m_ of the wild-type and each mutant µPlm were determined. The catalytic efficiency (Kcat/Km) was calculated according to the active enzyme concentration.

### Inhibition by α2-AP

Kinetic measurement of *Kcat* and Km was performed in the presence of increasing concentration of α2-AP, and IC50 was presented in the ratio to wild type, rather than absolute values, due to the variation of kinetic data in wild type from different batches. All experiments were done at 37 °C in 50 mM Tris–HCl, 100 mM NaCl, pH7.4.

### Homology modeling and molecular dynamics (MD) simulation

The µplasmin: α2-AP complex was constructed by homology modeling, starting from the crystal structures for μplasmin (PDB code: 1BML)^53^ and α2-AP (PDB code: 2R9Y)^54^. These two structures are superimposed to the crystal structure of Trypsin:antiTrypsin complex (PDB ID:1OPH^33^) to form the complex. The crystal structure of 2R9Y misses the C-terminal residues, therefore we use I-TASSER server^55^ to build the missing residues 465–491. The protein complex was then solvated in a rhombic dodecahedron solvent box of water molecules represented by the TIP3P^56^ model, and the size of the simulation unit cell was determined to be at least 10 Å away from any atom of the proteins. Counter ions (K^+^ and Cl^−^) were added to ensure electrostatic neutrality corresponding to an ionic concentration of ~ 150 mM. All protein covalent H-bonds were constrained with the LINCS^57^ algorithm. and long-range electrostatic interactions are treated with the particle-mesh Ewald^57^ method with a real-space cutoff of 10 Å. Parallel simulations are performed simultaneously using GROMACS 4.6^58^ in CHARMM36a1 force fields^59^. The system was minimized using the steepest decent algorithm to remove the bad contacts, and then gradually heated to 300 K at a constant volume over 1 ns, using harmonic restraints with a force constant 1,000 kJ/(mol Å^2^) on heavy atoms of both proteins and nucleotides. Over the following 5 ns of simulations at constant pressure (1 atm) and temperature (300 K), the restraints were gradually released. The systems were equilibrated for an additional 10 ns without positional restraints. A Parrinello–Rahman barostat^60^ was used to keep the pressure constant, while a V-rescale thermostat with a time step of 2 fs was used to keep the temperature constant. The system was simulated for 100 ns, with snapshots recorded every 20 ps.

## Supplementary information

Supplementary information
