# Supplementary material for: Selection of mutant µplasmin for amyloid-β cleavage in vivo
Source: Sci Rep. 2020 Jul 21;10:12117. doi: 10.1038/s41598-020-69079-8 (PMC7374754; doi:10.1038/s41598-020-69079-8)

**Supplemental Figures**

**Fig. S4: SDS-PAGE and Superdex 75 profiles.**

After refolding and concentration by ultrafiltration, µPlg wild-type and mutants were purified on a Superdex 75 SEC column as described^47^. **a** An example of the wild-type and 5 alanine mutants. In the SEC graph, the first peak is the unfolded aggregates, and the second peak (red arrows) is the refolded peak. A non-reduced SDS-PAGE of the purified proteins is shown in the insert. **b-1.** SDS-PAGE of 26 samples of the purified alanine mutants along with wild-type human and mouse samples. M is a molecular weight marker. 1. wild type; 2. W761A; 3. G762A; 4. R719A; 5. G695A; 6. G739A; 7. G739A; 8. G739A; 9. G764A; 10. T688A; 11. T688A; 12. L626A; 13. G693A; 14. K645A; 15. V624A; 16. H621A; 17. T691A; 18. V720A; 19. R610A; 20. Mouse-wild; 21. G690A; 22. L696A; 23. D740A; 24. F692A; 25. S760A; 26. L763A; 27. R582A; 28. P642A. **b-2.** SDS-PAGE of purified F587 mutants. 1. F587H; 2. F587I; 3. F587K; 4. F587L; 5. F587M; 6. F587N; 7. F587P; 8. F587Q; 9. F587R; 10. F587S; 11. F587T; 12. F587Y; 13. F587W; 14. F587V; 15. Wild-type (F); 16. F587C; 17. F587D; 18. F587E; 19. F587G. **c.** c-1 to c-4 are Superdex 75 purification profile and SDS-PAGE of F587 saturation mutagenesis proteins. c-1, 1. Wild-type; 2. F587A; 3. F587C; 4. F587D; 5. F587E; 6. F587G. c-2, 7. F587H; 8. F587I; 9. F587K; 10. F587L; 11. F587M; 12.F587N. c-3, 13. F587P; 14. F587Q; 15. F587R; 16. F587S; 17. F587T; 18. F587V. C-4, 19. F587W; 20. F587Y.


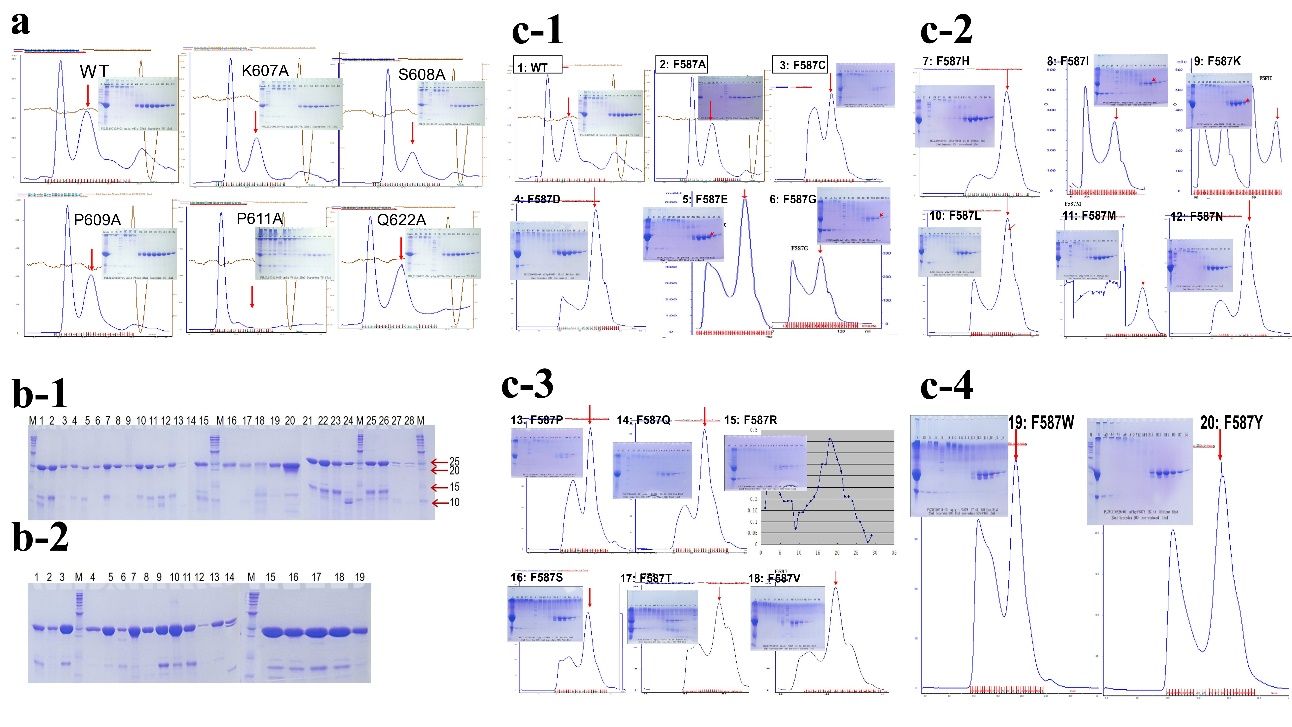


**Fig. S6:** **μPlm-α2-AP complex formation**.

**a** Human µPlm and α2-AP. M. Molecular weight marker, standard molecular weight is labeled on the left, and molecular weight (KD) of the protein fragments is labeled on the right. 1. Human α2-AP, 50 KD; 2. Human µPlg, 27 KD; 3, Human µPlm; 4, Human µPlg + α2-AP; 5, Human µPlm + α2-AP. **b** Mouse µPlm and α2-AP. Standard molecular weight is labeled on the left, and molecular weight (KD) of the protein fragments is labeled on the right. 1-6, reduced SDS-PAGE; 7-12, non-reduced SDS-PAGE. 1. Mouse µPlm, 28 KD; M, Molecular weight marker, labeled on the left; 2, Mouse α2-AP, 52 KD; 3, Mouse µPlm + Mouse α2-AP; 4. Mouse µPlm; 5, Mouse α2-AP; 6, Mouse µPlm + Mouse α2-AP; 7. Mouse µPlm; 8, Mouse α2-AP; 9, Mouse µPlm + Mouse α2-AP; 10. Mouse µPlm; 11, Mouse α2-AP; 12, Mouse µPlm + Mouse α2-AP. Reaction time, 1-3 and 7-9, 2 minutes; 4-6 and 10-12, 5 minutes. Urokinase (1:20) was used to activate µPlg in lanes 3, 4, 6, and 7; while staphylokinase (SAK, 18.5 KD, 1:1) was use to activate µPlg in lanes 9, 10, and 12. Lanes 13-16 were performed in a separate experiment. 13, Mouse µPlg; 14, Mouse µPlm; 15, Mouse µPlm + Mouse α2-AP; 16, Mouse µPlg + Mouse α2-AP. **c** Schematic presentation of the reaction between human α2-AP and µPlm shown in Fig. 4A. The red arrow from µPlm to R364 of human mature α2-AP illustrate the nucleophilic attach of the active site serine of µPlm toward the α2-AP substrate at the R364 (P1) position; **d** Schematic presentation of the reaction between mouse α2-AP and µPlm shown in Fig. 4B. The red arrow from µPlm to K148 of α2-AP illustrate the nucleophilic attach of the active site serine of µPlm toward the α2-AP substrate at the K148 (P1) position.


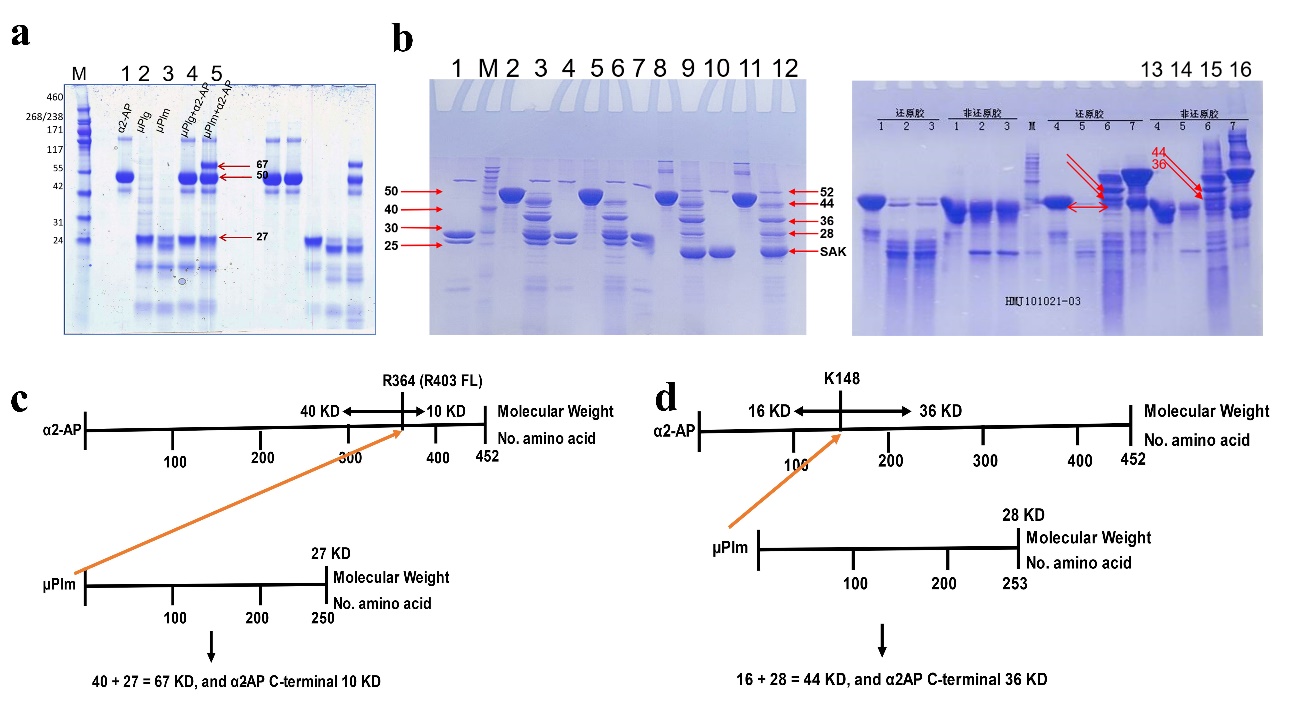

Supplement: Supplementary file 1 — Supplementary information [file 41598_2020_69079_MOESM1_ESM.docx]
